# Human Serum Albumin Nanoparticles for Use in Cancer Drug Delivery: Process Optimization and *In Vitro* Characterization

**DOI:** 10.3390/nano6060116

**Published:** 2016-06-15

**Authors:** Nikita Lomis, Susan Westfall, Leila Farahdel, Meenakshi Malhotra, Dominique Shum-Tim, Satya Prakash

**Affiliations:** 1 Biomedical Technology and Cell Therapy Research Laboratory, Department of Biomedical Engineering, 3775 University Street, Montreal, QC H3A 2B4, Canada; nikita.lomis@mail.mcgill.ca (N.L.); susan.westfall@mail.mcgill.ca (S.W.); leila.farahdel@mail.mcgill.ca (L.F.); 2 Division of Experimental Medicine, 1110 Pins Avenue, Montreal, QC H3A 1A3, Canada; 3 Department of Microbiology, Immunology and Infectious Diseases, CHU St. Justine Research Center, University of Montreal, 3175 Cote-Ste-Catherine, Montréal, QC H3T 1C5, Canada; meenakshi.malhotra@mail.mcgill.ca; 4 Division of Cardiac Surgery and Surgical Research, Royal Victoria Hospital, 1001 Boulevard Décarie, Montréal, QC H4A 3J1, Canada; dominique.shum-tim@muhc.mcgill.ca

**Keywords:** human serum albumin, nanoparticles, paclitaxel, cancer, MCF-7

## Abstract

Human serum albumin nanoparticles (HSA-NPs) are widely-used drug delivery systems with applications in various diseases, like cancer. For intravenous administration of HSA-NPs, the particle size, surface charge, drug loading and *in vitro* release kinetics are important parameters for consideration. This study focuses on the development of stable HSA-NPs containing the anti-cancer drug paclitaxel (PTX) via the emulsion-solvent evaporation method using a high-pressure homogenizer. The key parameters for the preparation of PTX-HSA-NPs are: the starting concentrations of HSA, PTX and the organic solvent, including the homogenization pressure and its number cycles, were optimized. Results indicate a size of 143.4 ± 0.7 nm and 170.2 ± 1.4 nm with a surface charge of −5.6 ± 0.8 mV and −17.4 ± 0.5 mV for HSA-NPs and PTX-HSA-NPs (0.5 mg/mL of PTX), respectively. The yield of the PTX-HSA-NPs was ~93% with an encapsulation efficiency of ~82%. To investigate the safety and effectiveness of the PTX-HSA-NPs, an *in vitro* drug release and cytotoxicity assay was performed on human breast cancer cell line (MCF-7). The PTX-HSA-NPs showed dose-dependent toxicity on cells of 52%, 39.3% and 22.6% with increasing concentrations of PTX at 8, 20.2 and 31.4 μg/mL, respectively. In summary, all parameters involved in HSA-NPs’ preparation, its anticancer efficacy and scale-up are outlined in this research article.

## 1. Introduction

Cancer is one of the leading causes of mortality and morbidity across the world. To date, the most common treatments for cancer include radiation therapy, chemotherapy and surgery [1]. However, their benefits are outnumbered by their disadvantages, such as renal toxicity, hepatic toxicity or lower availability of the drug at the target site [2]. These problems can be addressed by using a target-specific and biocompatible drug delivery vehicle, such as human serum albumin [3,4,5]. Human serum albumin (HSA) is the most abundant protein found in the human body with a molecular weight of 66.5 kDa. It is produced by the liver and has a half-life of around 19 days [6]. As revealed by X-ray structure analysis, the structure of HSA comprises of three domains: I, II and III. Each of these domains consists of two subdomains (Ia, Ib, IIa, IIb, IIIa and IIIb), which are arranged together to form binding sites on the HSA molecules [7]. HSA can bind to metabolic substrates, as well as therapeutic drugs, which include hydrophobic as well as hydrophilic drugs. HSA-NPs are formed by the aggregation of HSA molecules in solution forming intermolecular disulfide bonds [8]. The properties of HSA-NPs include biocompatibility, biodegradability and non-immunogenicity [4,9]. The target specificity of HSA for the glycoprotein60 (gp60) receptor present on the surface of cancer cells, allows the delivery of various anti-cancer drugs, such as docetaxel [10], paclitaxel [11] and noscapine [4], without inducing an immune response [5,9]. Paclitaxel is an anti-cancer drug, commercially available as Taxol^®^, and has been widely used as a chemo-therapeutic agent for the treatment of different cancer types, such as breast, ovarian and lung cancer [12,13]. Due to the toxic effects of this formulation on normal cells, Paclitaxel was used in combination with HSA-NPs (Abraxane^®^) for site-specific delivery [12,14,15]. This has led to improved tumor targeting by enhancement of the enhanced permeability and retention (EPR) effect as opposed to administration of free drugs [15].

The retention of these colloidal drug delivery systems within the body is highly influenced by the particle’s size, physical stability and surface characteristics [16]. It is known that nanoparticles in the size range 10–100 nm enter the lymphatic capillaries and undergo clearance [17]. Furthermore, particles in the size range of 250 nm–1 μm are identified by macrophages and removed by the reticuloendothelial system (RES) by the process of opsonization. This is a mechanism by which macrophages or monocytes identify and remove target cells or particles from the body by binding to them [17,18]. A lower surface curvature of the nanoparticles also lowers their chances of opsonization. This process is also influenced by the zeta potential of the particles. Negatively-charged particles prevent nanoparticle aggregation, whereas positively-charged particles promote binding to opsonin molecules, leading to their removal from blood circulation [19]. Thus, it is essential to control the particle size and zeta potentials of HSA-NPs to prevent their removal and ensure maximum efficacy.

In order to develop HSA-NPs containing paclitaxel, the emulsion-solvent evaporation method is the most reliable for the production of nanoparticles with a smaller size, a lower polydispersity index, reproducibility and potential for scale up [20]. The procedure is less complex, less time consuming and involves less use of chemicals for the preparation of HSA-NPs as compared to the pH coacervation technique or the microfluidics approach [21]. A high pressure homogenizer is commonly used for the breakdown of particles by the generation of high shear, which disperses the hydrophobic drug into the HSA solution, forming homogeneously-dispersed nanoparticles [14]. This technique was first demonstrated by Desai *et al.* for the production of paclitaxel-bound HSA-NPs and can be used for improving the water solubility of most hydrophobic drugs [14]. Kim *et al.* later used this technique for the preparation of curcumin containing HSA-NPs [22].

In this study, the main aim was to develop and optimize the preparation process of HSA-NPs, following the emulsion-solvent evaporation method, in order to prepare reproducible and stable paclitaxel (PTX)-HSA-NPs with a particle size between 100 and 200 nm. The yield and encapsulation efficiencies of the particles were higher in comparison to other studies. By the optimization of the parameters, a high particle yield and high drug encapsulation efficiency was obtained. The nanoparticles were produced by high pressure homogenization (HPH) by optimizing parameters, such as the HSA concentration, organic solvent concentration, homogenization pressure, number of homogenization cycles and the Paclitaxel (PTX) concentration [22]. These parameters were found to influence the final particles size, surface charge and morphology of the nanoparticles. The surface morphology of the nanoparticles was analyzed by using scanning electron microscopy (SEM). Further, to evaluate the effectiveness of the optimized parameters, different concentrations of PTX were added to prepare PTX-HSA-NPs. The safety effectiveness of the PTX-HSA-NPs was tested by performing an *in vitro* drug release and cytotoxicity assay using human breast cancer cell line (MCF-7).

## 2. Results

### 2.1. Nanoparticles Optimization and Preparation

In this study, various parameters crucial in the preparation of PTX-HSA-NPs of sizes 100–200 nm were optimized using a high pressure homogenizer. The working of the high pressure homogenizer is based on the principal that applying a very high pressure (5,000–60,000 psi) to the emulsion passed through a homogenization valve breaks the emulsion into nano-sized emulsion droplets [14,23,24]. Evaporating the organic solvent from the emulsion leads to the formation of nanoparticles.

#### 2.1.1. Effect of Homogenization Pressure on Nanoparticles

**Figure 1:**
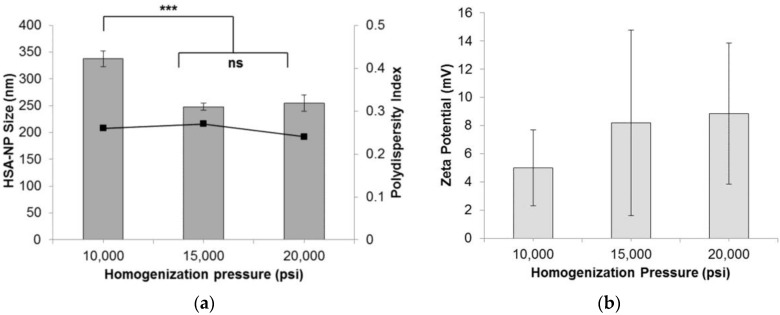
Effect of homogenization pressure (psi) on the (**a**) human serum albumin-nanoparticle (HAS-NP) size (mean ± SD, *n* = 10), represented as columns, and polydispersity index, represented as line; (**b**) zeta potential of HSA-NPs (mean ± SD, *n* = 10), represented as columns, prepared from 20 mg/mL starting HSA concentration, 12 homogenization cycles and a chloroform concentration of 3% *v*/*v* of starting HSA solution (*** *p* < 0.0001; ns = not significant).

To form HSA-NPs, the homogenization pressure was optimized by applying 10,000 psi, 15,000 psi and 20,000 psi, to the starting emulsion. The starting HSA concentration was 20 mg/mL with a chloroform concentration of 3% *v*/*v*, and 12 homogenization cycles were applied. Results suggested that on increasing the pressure above 10,000 pounds per square inch (psi), the average size (mean ± standard deviation (SD)) of the HSA-NPs was reduced from around 337.7 ± 14.8 nm down to 248.2 ± 6.6 nm at 15,000 psi and 254.7 ± 15.5 nm at 20,000 psi (Figure 1a). The HSA-NP sizes at 15,000 psi and 20,000 psi were not significantly different. The zeta potentials of the nanoparticles were around 5–8 mV (Figure 1b). The polydispersity index (PDI) for all of the samples was less than 0.3. For the next experiments, the homogenization pressure was set to 20,000 psi to obtain smaller-sized nanoparticles [24].

#### 2.1.2. Effect of Homogenization Cycles on Nanoparticles

**Figure 2:**
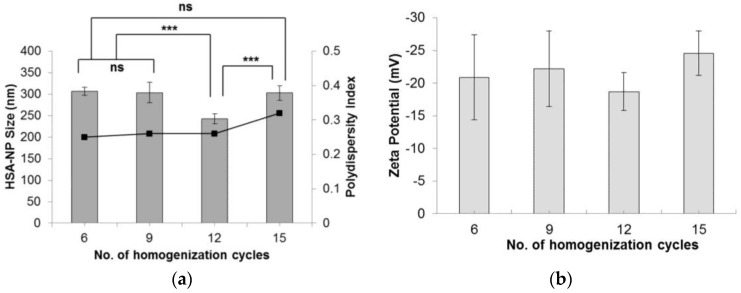
Effect of varying the number of homogenization cycles on the (**a**) HSA-NP size (mean ± SD), *n =* 10), represented as columns, and polydispersity index, represented as line; (**b**) zeta potential of HSA-NPs (mean ± SD, *n =* 10), represented as columns, prepared from a 20 mg/mL starting HSA concentration, 20,000 psi pressure with a chloroform concentration of 3% *v*/*v* of starting HSA solution (*** *p* < 0.0001; ns = not significant).

The effect of the number of homogenization cycles on HSA-NP size was evaluated by applying 6, 9, 12 and 15 cycles. Starting with a 20 mg/mL HSA concentration, 3% *v*/*v* chloroform and 20,000 psi pressure, results indicated that on applying 12 homogenization cycles, HSA-NPs of an average size 250 ± 11.7 nm were formed, which was significantly lower than the other samples (Figure 2a). The zeta potential of the nanoparticles was −18 ± 2.9 mV. Comparing the particle size obtained by applying 6, 9 and 15 homogenization cycles did not show any significant difference. Furthermore, there was no significant difference in the zeta potential values (Figure 2b). The PDI of the particles was less than 0.4. Increasing the number of homogenization cycles combined with the applied pressure led to a greater reduction in particle size [25].

#### 2.1.3. Effect of HSA Concentration on Nanoparticle Size

**Figure 3:**
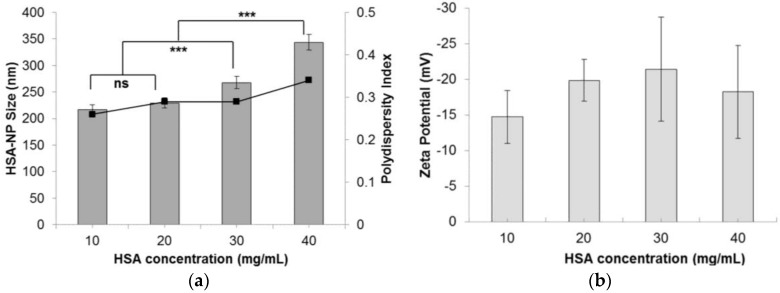
Effect of the starting HSA concentration (mg/mL) on the (**a**) HSA-NP size (mean ± SD, *n* = 10), represented as columns, and polydispersity index, represented as line; (**b**) zeta potential of HSA-NPs (mean ± SD, *n* = 10), represented as columns, prepared with a chloroform concentration of 3% *v*/*v* of starting HSA solution, 12 homogenization cycles and 20,000 psi homogenization pressure (*** *p* < 0.0001; ns = not significant).

The nanoparticle size was also influenced by varying the starting HSA concentration. As the HSA concentration was increased from 10 mg/mL to 40 mg/mL, the HSA-NP size increased from 216.6 ± 9.8 nm to 343.6 ± 14.6 nm, respectively, as represented in (Figure 3a). Consequently, the zeta potential of the HSA-NPs varied from −14.7 ± 13.7 mV to −18.3 ± 6.5 mV (Figure 3b). For the 10 mg/mL HSA concentration, the PDI was approximately 0.26, which was lower than that of other concentrations. It was also noted that on increasing the HSA concentration in solution, the PDI of the solution also increased.

#### 2.1.4. Effect of Chloroform on Nanoparticles

**Figure 4:**
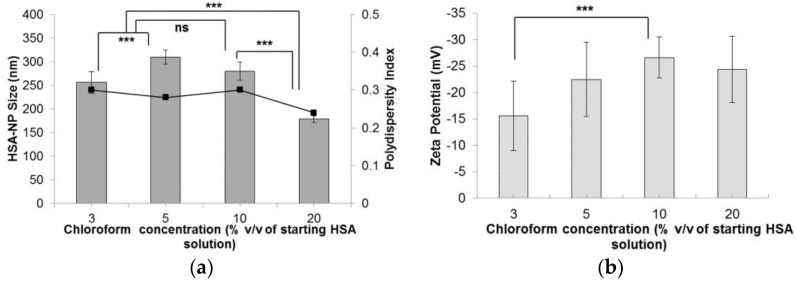
Effect of chloroform concentration (% *v*/*v* of starting HSA solution) on the (**a**) HSA-NP size (mean ± SD, *n* = 10), represented as columns, and polydispersity index, represented as line; (**b**) zeta potential of HSA-NPs (mean ± SD, *n* = 10), represented as columns, prepared from a 10 mg/mL HSA concentration, 20,000 psi homogenization pressure and 12 homogenization cycles (*** *p* < 0.0001; ns = not significant).

Following the optimization of the HSA starting concentration, the experimental conditions were set to a 10 mg/mL HSA concentration, 20,000 psi homogenization pressure and 12 homogenization cycles. The effect of chloroform concentration (% *v*/*v*) in the starting HSA solution was investigated. On increasing the chloroform concentration from 1% *v*/*v* to 20% *v*/*v*, the size of the HSA-NPs decreased from 256.1 ± 22 nm down to 178.4 ± 6.7 nm (Figure 4a). The zeta potential of the HSA-NPs decreased from −15.5 ± 6.6 mV down to −24.3 ± 6.3 mV, respectively (Figure 4b). The PDI of the nanoparticle solution was also lowered from 0.30 down to 0.24, respectively.

#### 2.1.5. Effect of Chloroform-Ethanol Concentration on Nanoparticles

**Figure 5:**
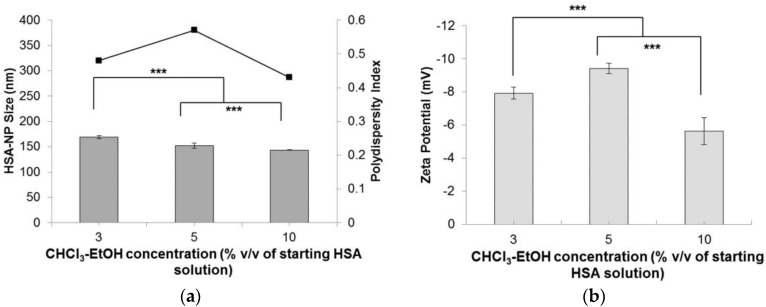
Effect of chloroform-ethanol concentration (% *v/v* of starting HSA solution) on the (**a**) HSA-NP size (mean ± SD, *n* = 10), represented as columns, and polydispersity index , represented as line; (**b**) zeta potential of HSA-NPs (mean ± SD, *n* = 10), represented as columns, prepared from a 10 mg/mL HSA concentration, 20,000 psi homogenization pressure and 12 homogenization cycles (*** *p* < 0.0001; ns = not significant).

Starting with a HSA concentration of 10 mg/mL, 20% *v/v* chloroform, 20,000 psi homogenization pressure and applying 12 homogenization cycles resulted in the formation of HSA-NPs of an average size of 178.4 ± 6.7 nm and a zeta potential of −24.3 ± 6.3 mV. However, in order to reduce the use of the 20% *v/v* chloroform concentration, for the preparation of HSA-NPs of size less than 200 nm, chloroform was mixed with ethanol in a ratio of 94:6. Using the optimized experimental conditions, instead of using 20% *v/v* chloroform, the effect of CHCl_3_-EtOH (3% *v/v*, 5% *v/v* and 10% *v/v* of HSA solution) on the HSA-NP size and surface charge was investigated. Results suggested that increasing the concentration of CHCl_3_-EtOH, from 3% *v/v* to 10% *v/v*, in the HSA emulsion, reduced the size of the HSA-NPs from 169.1 ± 2.6 nm down to 143.4 ± 0.7 nm, respectively (Figure 5a). There was a significant difference in the zeta potentials of the nanoparticles, which varied between approximately −5 and −10 mV (Figure 5b). However, the PDI of the HSA-NPs increased to greater than 0.4.

#### 2.1.6. Effect of Paclitaxel Addition in Nanoparticles

**Figure 6:**
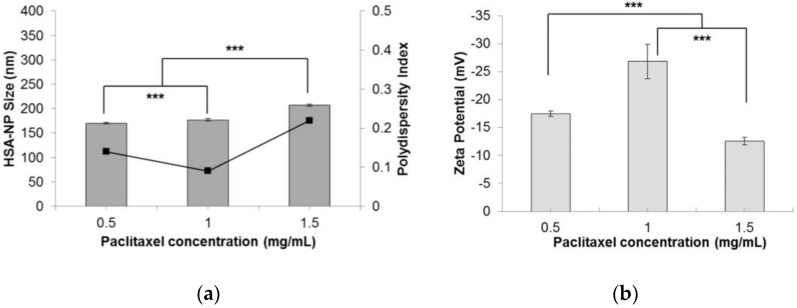
Effect of paclitaxel (PTX) concentration (mg/mL) on the (**a**) HSA-NP size (mean ± SD, *n* = 10), represented as columns, and polydispersity index, represented as line; (**b**) zeta potential of HSA-NPs (mean ± SD, *n* = 10), represented as columns, prepared from a 10 mg/mL starting HSA concentration, a 3% *v*/*v* CHCl_3_-EtOH concentration, 20,000 psi homogenization pressure and 12 cycles of homogenization (*** *p* < 0.0001).

Finally, to test the effectiveness of the optimized conditions, different concentrations of paclitaxel (0.5, 1 and 1.5 mg/mL) were added to the starting HSA solution, to form PTX-HSA-NPs. A 10 mg/mL HSA concentration, 3% *v/v* CHCl_3_-EtOH, 20,000 psi homogenization pressure and 12 cycles of homogenization were applied. Results suggested that by increasing the amount of PTX added to the starting solution, the size of PTX-HSA-NPs increased from 170.2 ± 1.4 nm to 207.5 ± 2.4 nm, respectively (Figure 6a). The PDI also increased from 0.14 to 0.22. This was contradictory to the results obtained by Desai *et al.* in a study that suggested that increasing the drug concentration in the nanoparticle solution reduces the particle size [14]. The zeta potential of the PTX-HSA-NPs was not affected by varying the PTX concentration (Figure 6b). The PTX-HSA-NPs prepared from 1 mg/mL PTX concentration was the most optimized condition with an HSA-NP size of 177.1 ± 2.5 nm, a zeta potential of −26.8 ± 3.1 mV and PDI 0.09.

A complete summary of the optimized parameters and the resulting particle sizes, zeta potentials and polydispersity index (PDI) is represented in Table 1.

### 2.2. Size, Stability, Morphology, Yield and Encapsulation Efficiency of HSA-NPs

**Figure 7:**
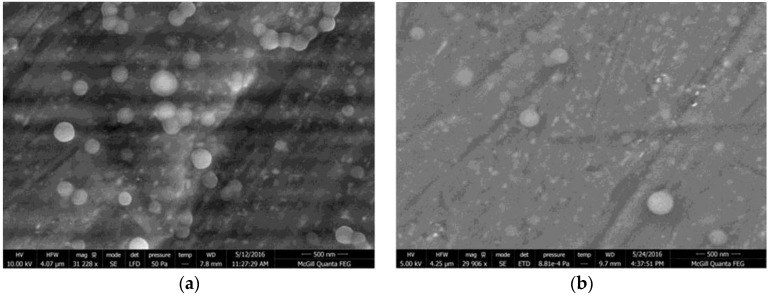
(**a**) Scanning electron microscope (SEM) image of HSA-NPs of a size of 143.4 ± 0.7 nm and a charge of −5.6 ± 0.8 mV, prepared from optimized experimental conditions (scale = 500 nm). (**b**) SEM image of paclitaxel human serum albumin nanoparticles (PTX-HSA-NPs) of a size of 177.1 ± 2.5 nm and a charge of −26.8 ± 3.1 mV, prepared from optimized experimental conditions with a 1 mg/mL starting paclitaxel (PTX) concentration (scale = 500 nm).

PTX-HSA-NPs were prepared by following the optimized experimental conditions. The size and morphology of the nanoparticles were observed by SEM, as shown in Figure 7. The SEM images displayed HSA-NPs and PTX-HSA-NPs of an average size of less than 200 nm with a round surface morphology.

The yield of the HSA-NPs, prepared from optimized parameters, was approximately 94% and for the PTX-HSA-NPs, prepared from different PTX concentrations, varied between approximately 92% and 94%. These results have been summarized in Table 2. It was noted that the drug encapsulation efficiency increased with an increase in the paclitaxel concentration. For PTX-HSA-NPs prepared from concentrations of 0.5 mg/mL PTX, 1 mg/mL PTX and 1.5 mg/mL PTX, the encapsulation efficiency was approximately 82%, 94% and 98%, respectively.

### 2.3. In Vitro Drug Release Study

**Figure 8:**
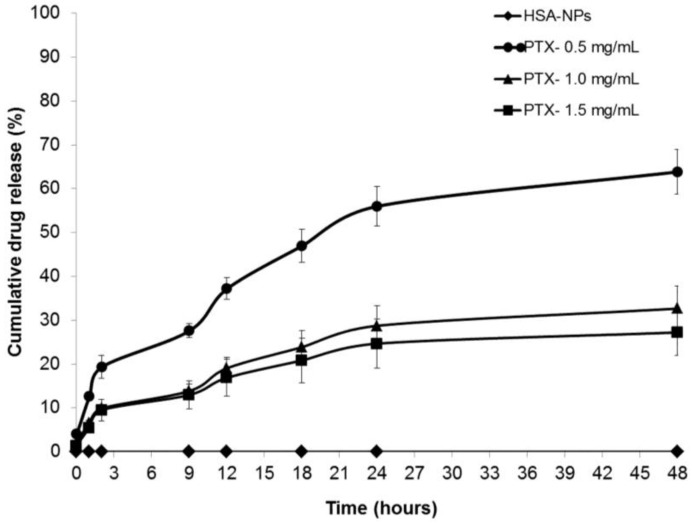
Cumulative drug release (mean ± SD %, *n* = 3) profiles of PTX-HSA-NPs prepared from different PTX starting concentrations (0.5, 1 and 1.5 mg/mL) compared to HSA-NPs (without PTX) displaying the cumulative release of PTX from PTX-HSA-NPs over time intervals of 0, 1, 2, 9, 12, 18, 24 and 48 h.

The drug release profiles of the PTX-HSA-NPs for three different samples prepared from 0.5, 1 and 1.5 mg/mL starting PTX concentrations were analyzed. The PTX-HSA-NPs samples were labeled PTX-0.5 mg/mL, PTX-1.0 mg/mL and PTX-1.5 mg/mL. The PTX-HSA-NPs prepared from the optimized conditions were dispersed in 5 mL phosphate buffered saline (PBS) (pH 7.4) at 120 rpm at 37 °C. This method of *in vitro* drug release in which the different amounts of drug released into the dispersion medium over a period of time can be measured and has previously been demonstrated in the literature [4,26,27]. The amount of PTX released into the nanoparticle solution at fixed intervals over a period of 48 h was determined and compared to the control sample containing HSA-NPs without paclitaxel, as shown in Figure 8. Approximately 63.8% ± 6.8%, 32.6% ± 5.1% and 27.2% ± 5.2% of the drug were released within 48 h from the PTX-HSA-NPs prepared from 0.5 mg/mL, 1 mg/mL and 1.5 mg/mL PTX concentrations, respectively. The drug release became much slower after 48 h for all three samples.

### 2.4. Cell Culture and In Vitro Cell Viability

**Figure 9:**
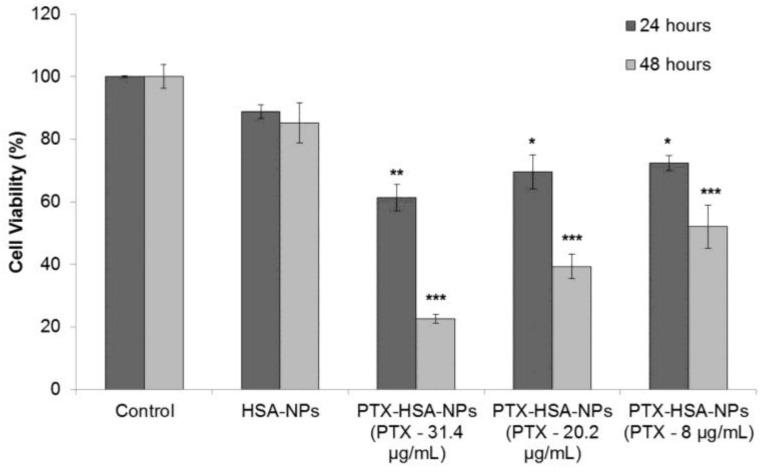
Dye 3-(4,5-dimtheylthiazol-2-yl)-2,5-diphenltetrazoliumbromide (MTT) assay measuring the effect of PTX-HSA-NPs, prepared from different starting PTX concentrations, on the cell viability of human breast cancer cell line (MCF-7) as compared to HSA-NPs at 24 and 48 h, respectively. The graph shows a representative result of (*n* = 3) mean ± S.D. * *p* < 0.05, ** *p* < 0.01 and *** *p* < 0.001 were considered significant based on Tuckey’s *post hoc* analysis.

For evaluating the safety and efficacy of PTX-HSA-NPs, MCF-7 breast cancer cells were cultured in 96-well plates at an initial concentration of 5,000 cells/well in fresh medium. After 24 h of culture, the medium was replaced with HSA-NPs and PTX-HSA-NPs for 24 and 48 h. The HSA-NP concentration in solution was 0.2 mg/mL. The final concentrations of PTX in the three samples of PTX-HSA-NPs (prepared with starting PTX concentrations of 0.5, 1 and 1.5 mg/mL) were 8, 20 and 31.4 μg/mL, respectively, in the three treatment groups. The cell viability of the PTX-HSA-NPs and HSA-NPs treatments was investigated. Results suggested that the HSA-NPs treatment group showed a cell viability of 88.8% ± 2.2% at 24 h and 85.3% ± 6.4% at 48 h. The cell viability of the PTX-HSA-NPs treatment group, containing PTX-31.4 μg/mL, was 61.3% ± 4.2% at 24 h, which reduced to 22.6% ± 1.4% at 48 h. For the PTX-HSA-NPs treatment group, containing PTX-20 μg/mL, the cell viability was 69.5% ± 5.4% at 24 h, which reduced to 39.3% ± 3.9% at 48 h. For the PTX-HSA-NP treatment group containing PTX-8 μg/mL, the cell viability was 72.4% ± 2.4% at 24 h, which was reduced to 52.0% ± 6.8% at 48 h. These results are shown in Figure 9.

**Figure 10:**
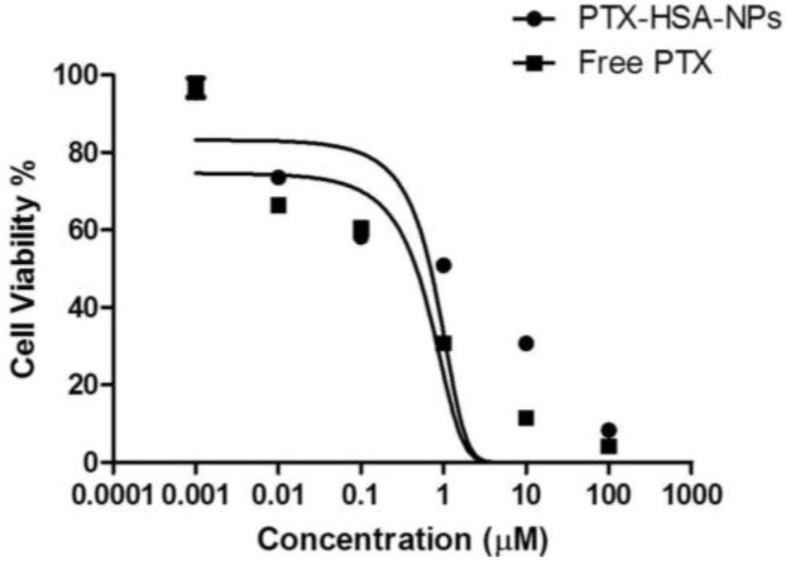
Cell viability for PTX-HSA-NPs and free PTX in MCF-7 breast cancer cells with the mean ± 95% confidence interval. The graph was fitted using a nonlinear regression model in GraphPad Prism software version 5.01 (GraphPad Software Inc., La Jolla, CA, USA) and IC_50_ was calculated using the dose-response inhibitory equation.

A dose-response study was performed on the optimized formulation of PTX-HSA-NPs and the free PTX by treatment with MCF-7 breast cancer cells in order to evaluate the efficacy of the optimized PTX-HSA-NPs. The proliferation of the MCF-7 cells was found to be inhibited on treatment with the PTX-HSA-NPs and compared to the free PTX treatment group, as shown in Figure 10. The half maximal inhibitory concentration (IC_50_) of PTX for the PTX-HSA-NPs was calculated to be 4.9 μM and 2.1 μM for free PTX. The IC_50_ is the concentration of PTX or an equivalent of PTX required for killing 50% of the viable MCF-7 cells.

## 3. Discussion

The incidence of cancer is rapidly increasing throughout the world. It is thus important to develop more effective and efficient nanoparticle systems, such as HSA-NPs, for specific targeting of anticancer drugs like paclitaxel [13,28]. This study demonstrates the development of stable HSA-NPs using the emulsion-solvent evaporation method. This method is commonly used for improving the solubility of hydrophobic drugs by attachment to HSA-NPs or other polymeric nanoparticles [29,30]. As compared to other preparation methods, such as the pH coacervation method, this process is less complex, less time consuming and suitable for scale up [31]. A high pressure homogenizer was used for the preparation of HSA-NPs and PTX-HSA-NPs of sizes in the range of 100–200 nm. High pressure homogenization is a technique used for the production of nano-sized emulsions [25]. In this study, it was used to disperse paclitaxel, a water-insoluble anti-cancer drug, into the aqueous HSA solution. This emulsion on being subjected to repeated homogenization cycles is broken down into nano-sized emulsions, which ultimately form nanoparticles [24]. This technique has also been applied to other therapeutic drugs, such as curcumin, doxorubicin and pirarubicin-paclitaxel for use in cancer therapy [22,32,33]. It allows the dispersion of hydrophobic drug formulations into the aqueous HSA solution by facilitating their binding to the hydrophobic cavity on the HSA molecule [34,35]. Thus, the potential of such a methodology for the development of reproducible and stable drug containing HSA-NPs is greatly enhanced.

We optimized various parameters, such as the HSA starting concentration, organic solvent concentration, homogenization pressure, number of homogenization cycles and PTX concentration. Three different starting concentrations of PTX (0.5, 1 and 1.5 mg/mL) were tested by adding to the preparative HSA solution, which resulted in the formation of PTX-HSA-NPs. From the first experiment, results suggested that increasing the homogenization pressure from 10,000 psi to 20,000 psi led to a decrease in the HSA-NP size. This trend is expected since in order to reduce the particle size, it is necessary to overcome the minimum pressure, known as the Laplace pressure [23]. The zeta potential of the particles showed a positive charge with a high standard deviation, which indicated the instability of the HSA-NPs (Figure 1). Thus, further optimization was necessary to develop stable HSA-NPs, which was accomplished in the subsequent steps. It was observed that increasing the number of homogenization cycles led to a reduction in the size of the HSA-NPs. This was because, on increasing the applied homogenization pressure and number of homogenization cycles, the emulsion was repeatedly subjected to high shear, which further reduced the size of the emulsion droplets into nano-sized droplets [23,36]. The surface charge on the particles was not significantly different due to the high standard deviations (Figure 2). Increasing the HSA concentration led to an increase in the size of the HSA-NPs with a decrease in the zeta potential (Figure 3). HSA is a negatively-charged molecule, thus, increasing the amount of HSA in the starting solution leads to greater formation of intermolecular disulfide bonds. This further causes higher protein aggregation and the formation of larger-sized HSA-NPs with a more negative zeta potential [37]. The effect of chloroform in the solution was also investigated. It was found that increasing the chloroform concentration led to a decrease in the size of the HSA-NPs (Figure 4). The nanoparticles with the smallest size were formed when the chloroform concentration in the starting solution was 20% *v*/*v* of the starting HSA solution. Increasing the amount of organic solvent in the reaction mixture provides a larger surface area for the emulsion, undergoing repeated homogenization at high pressure, to be reduced to smaller droplets due to high shear. However, in order to minimize the exposure to higher amounts of chloroform, it was replaced with chloroform-ethanol in the ratio of 94:6. It is known that different organic solvents have different effects on the size of the emulsion droplets [38]. It was noted that using 3% *v*/*v* CHCl_3_-EtOH in solution resulted in HSA-NPs of a size comparable to those formed using 20% *v*/*v* CHCl_3_. Increasing the concentration of CHCl_3_-EtOH in the HSA solution reduced the size of the HSA-NPs further (Figure 5) [39]. On evaporating the organic solvent, the nanoparticles are retained in solution. It is important to evaporate the chloroform slowly under reduced pressure, keeping the bath temperature at 40 °C, in order to prevent immediate aggregation of the nanoparticles.

Lastly, the effects of varying the paclitaxel concentration (0.5, 1 and 1.5 mg/mL on the size and surface charge of the PTX-HSA-NPs was investigated. It was noted that increasing the PTX concentration from 0.5 mg/mL to 1.5 mg/mL led to an increase in the particle size and a more negative zeta potential (Figure 6). The PDI of the nanoparticle solution was less than 0.2 in all of the samples. This result was contradictory to the results obtained by Desai *et al.*, who demonstrated that increasing the paclitaxel concentration leads to the formation of smaller-sized PTX-HSA-NPs [14]. It is possible that this variation resulted due to the difference in organic solvents used for the preparation of the PTX-HSA-NPs or due to the optimized conditions in this study. It was observed that the encapsulation efficiency of the PTX-HSA-NPs was approximately 82%, 94% and 98%, which was much higher than those obtained by other research groups. Zhao *et al.* and his team, who prepared paclitaxel-loaded HSA-NPs by a microfluidic technique and incorporating glutathione in HSA-NPs for additional stabilization, reported a maximum encapsulation efficiency of 11% for the PTX-HSA-NPs. Similarly, Gong *et al.* co-encapsulated paclitaxel with pirarubicin to enhance the anti-tumor effect of the formulation and reported encapsulation efficiency of around 80% for paclitaxel [33]. A high yield and high encapsulation efficiency allows greater entrapment of the drug molecules in the HSA-NP binding sites, and therefore, for lower nanoparticle concentrations, high drug release and cytotoxicity could be observed.

The *in vitro* drug release of PTX from the PTX-HSA-NPs at time intervals 0, 1, 2, 9, 12, 18, 24 and 48 h was studied in triplicates (Figure 8). This method of *in vitro* drug release has previously been demonstrated in the literature [4,26,27]. The PTX-HSA-NPs are dispersed in PBS at 37 °C and shaking conditions in order to simulate the dynamic *in vivo* conditions. This is an indicator of the controlled release behavior of PTX-HSA-NPs. It was observed that the PTX-HSA-NPs prepared from a PTX concentration of 0.5 mg/mL provided a burst release of approximately 37.2% ± 2.1% of the drug within 12 h. Despite this initial burst release, the cumulative release reached 63.1% ± 6.8% within 48 h in a consistent manner. The size of the nanoparticles was 170.2 ± 1.4 nm; the charge was −17.44 ± 0.5 mV; and the drug encapsulation efficiency was approximately 82%. [40]. The PTX-HSA-NPs prepared with a starting PTX concentration of 1.5 mg/mL released the drug slowly with only 27.2% ± 5.2% released in 48 h. Similarly, the cumulative release from PTX-HSA-NPs initially prepared from 1 mg/mL PTX concentration was 32.6% ± 5.1% at the end of 48 h. These results are comparable to a study using PTX-containing bovine serum albumin nanoparticles (BSA-NPs) showing an initial burst release and later consistency in the *in vitro* drug release [41]. It allows continuous targeting of the cancer cells with a decrease in cell viability over time, as opposed to complete release of the drug within 24 h. Contrarily, other studies demonstrate a quick cumulative release of approximately 80% drug from PTX-HSA-NPs within 12 h followed by a slow release [11]. This condition is not suitable, since the half-life of paclitaxel lies between 3 and 52 h, after which, the drug will be removed by hepatic clearance without eluting a pronounced toxic effect on the cancer cells [42].

The cytotoxicity of the formulations including HSA-NPs, as well as PTX-HSA-NPs was tested on MCF-7 human breast cancer cells. The MCF-7 cells treated with PTX-HSA-NPs were incubated under static conditions as opposed to the shaking conditions for measuring the *in vitro* drug release from PTX-HSA-NPs, because the cultured cells have enzymes that will subsequently degrade the nanoparticles over a period of time. However, the final goal is to test the PTX-HSA-NPs *in vivo*, which is under dynamic conditions. The cell viability on incubation with HSA-NPs was approximately 88.5% ± 2.2% at 24 h and 85.3% ± 6.4% at 48 h and, hence, did not reduce significantly (Figure 9). In the case of the PTX-HSA-NP formulations, the cells exhibited a concentration-dependent toxicity. Incubating the cells with the PTX-HSA-NPs with PTX concentrations of 31.4 μg/mL, 20.2 μg/mL and 8 μg/mL resulted in cell viability of approximately 61.3% ± 4.2%, 69.5% ± 5.4% and 72.4% ± 2.4%, respectively, at 24 h. The cell viability was reduced drastically to 22.6% ± 1.4%, 39.3% ± 3.9% and 52.1% ± 2.4%, respectively, at 48 h, as compared to the HSA-NPs. Assuming that approximately 62%, 32% and 27% of drug was released from the PTX-HSA-NPs containing 8 μg/mL, 20.2 μg/mL and 31.4 μg/mL of PTX, respectively, at 48 h, the expected cell viability is close in range with that measured using the dye 3-(4,5-dimtheylthiazol-2-yl)-2,5-diphenltetrazoliumbromide (MTT) assay. This study can be closely compared with a study in the literature by Bernaheu *et al.*, who have demonstrated the *in vitro* performance of Abraxane on human breast cancer cells (MCF-7) and human breast cancer cells (MDA-MB-231) cells using similar cell culture conditions as in our study. Their cytotoxicity results revealed that an Abraxane concentration of 100 μg/mL resulted in cell viabilities of approximately 55% and 23% for MCF-7 and MDA-MB-231 cells, respectively [43]. However, in our study, the optimized PTX-HSA-NPs with PTX 31.4 μg/mL cause a cytotoxic effect with only 22% cell viability at 48 h. Other studies in the literature have also studied the effect of Abraxane on non-small-cell lung cancer cells (A549) and prostate cancer cells (PC3) [44,45]. However, the results are not comparable due to the variation in experimental conditions and the difference in the *in vitro* cell study.

Lastly, the dose-dependent response of the optimized PTX-HSA-NPs was studied in comparison with free PTX on the MCF-7 breast cancer cell line. After statistical analysis, an IC_50_ of 4.9 μM for the optimized PTX-HSA-NPs and 2.1 μM for the free PTX was obtained. These values are lower than the >100 μM IC_50_ values for Abraxane when tested in the MCF-7 breast cancer cell, as observed in a study by Bernaheu *et al.* [43]. Another study in the literature has demonstrated IC_50_ values of 11.07 nM and 8.57 nM, for Abraxane and PTX, respectively, when tested in A549 non-small-cell lung cancer cells [45]. The calculated IC_50_ value of PTX was 12.4 μM when tested on 4T1 murine breast cancer cells [33]. On testing in MDA-MB-231 breast cancer cells, IC_50_ values for Abraxane and PTX were 2.7 nM and 2.5 nM, respectively, whereas in PC3 cells, 11.9 nM and 5.3 nM, respectively [44]. Thus, the IC_50_ values of the PTX-HSA-NPs and free PTX differ when evaluated in different cell lines.

Therefore, on comparing the *in vitro* performance of the optimized PTX-HSA-NPs with Abraxane and similar PTX-containing nanoparticle formulations at the given experimental conditions, from the above discussion, it can be concluded that this study demonstrates higher encapsulation efficiency, improved drug release characteristics and improved cytotoxicity. Therefore, the optimized PTX-HSA-NPs are anticipated to exhibit enhanced anticancer characteristics.

## 4. Materials and Methods

### 4.1. Reagents and Chemicals

Human serum albumin (lyophilized powder, ≥96%) was obtained from Sigma Aldrich (Oakville, ON, Canada). Paclitaxel (powder) was obtained from LC Laboratories (Woburn, MA, USA). Chloroform and all other chemicals were obtained from VWR International (Mississauga, ON, Canada).

### 4.2. High Pressure Homogenizer

The Avestin C-5 High Pressure Homogenizer (Avestin Inc., Ottawa, ON, Canada) was used for the preparation of HSA-NPs by the application of high pressure (5,000–60,000 psi) to break the HSA-containing emulsion into nano-sized emulsion droplets. The working solution volume for the preparation of HSA-NPs was 10 mL.

### 4.3. Process Optimization and Preparation of PTX-HSA-NPs 

The HSA-NPs were prepared by the emulsion-solvent evaporation method using a high pressure homogenizer. The starting HSA concentrations of 10 mg/mL, 20 mg/mL, 30 mg/mL and 40 mg/mL were prepared in 10 mL of deionized water. To the preparative HSA solution, chloroform (CHCl_3_) (3% *v*/*v*, 5% *v*/*v*, 10 and 20% *v*/*v*) was added to the starting HSA solution. On further optimization, the chloroform addition step was replaced by the addition of a mixture of chloroform and ethanol (EtOH) in the ratio of 94:6. The concentrations of CHCl_3_-EtOH in the starting HSA solution were 3%, 5% and 10% *v*/*v*. This emulsion was first subjected to primary homogenization for 3.5 min, using a hand-held D1000 Benchmark homogenizer (Benchmark Scientific, Edison, NJ, USA) followed by high pressure homogenization. The homogenization pressure (10,000 psi, 15,000 psi and 20,000 psi) was applied to the emulsion, and the number of homogenization cycles (6, 9, 12 and 15 cycles) was also optimized. The emulsion subjected to various homogenization cycles was passed through the homogenizer valve and collected through a connecting tube at the base of the assembly, thus forming nano-sized emulsion droplets.

For the preparation of paclitaxel-containing HSA-NPs, paclitaxel (0.5, 1 and 1.5 mg/mL) was dissolved in a 3% *v*/*v* CHCl_3_-EtOH mixture (94:6) and then mixed with the HSA solution volume. This emulsion was subjected to primary homogenization for 3.5 min using the hand-held D1000 Benchmark homogenizer. After primary homogenization, the emulsion was subjected to 12 cycles of high pressure homogenization at 20,000 psi pressure per cycle.

Following high pressure homogenization, the resulting colloidal solution was transferred to a round-bottomed flask and subjected to rotary evaporation at 90 rpm by applying a vacuum pressure of 400 mm Hg for 30 min at 40 °C. This process ensured complete removal of the organic solvent from the emulsion, which led to the formation of HSA-NPs.

### 4.4. Nanoparticles Size Measurement, Zeta Potential Analysis and Surface Morphology

The average particle size of the HSA-NPs, both with and without paclitaxel, was measured by dynamic light scattering (DLS) using a particle size analyzer (Brookhavens Instruments Corporation, Holtsville, NY, USA). The samples were diluted with deionized water and measured at a scattering angle of 90° and temperature of 25 °C. The polydispersity index (PDI) gave an estimate of the size distribution of the HSA-NPs. The zeta potential was measured by a zeta potential analyzer (Malvern Instruments, Worcestershire, UK) using electrophoretic laser Doppler anemometry. The size, shape and surface morphology of the HSA-NPs were examined by scanning electron microscopy (Hitachi S-4700 FE-SEM, Tokyo, Japan).

### 4.5. Yield and Encapsulation Efficiency of HSA-NPs

The yield of the HSA-NPs was measured by the UV-spectrophotometric method. A standard curve of HSA dissolved in a solution of phosphate buffered saline (PBS), containing 0.02% *v*/*v* Tween 20% *v*/*v* and 10% *v*/*v* acetonitrile, was used as a reference. The absorbance values for HSA were measured at 280 nm. For the calculation of the yield, the following equation was used.

Yield% = (weight of HSA in solution/initial weight of HSA used) × 100

To calculate the encapsulation efficiency of paclitaxel in HSA-NPs, the PTX-HSA-NPs were spin concentrated using Amicon centrifugal filters (Cedarlane, Burlington, ON, Canada) with a molecular weight cut off (MWCO) of 10,000 Da. This allowed the non-encapsulated paclitaxel drug to be eluted out in the collection tube. The concentration of non-encapsulated PTX was determined by the UV-spectrophotometric method. A standard curve of PTX in a mixture containing MeOH/PBS (30:70) and 1% *v*/*v* sodium dodecyl sulfate (SDS) was used as a reference [46]. The absorbance values were measured at 230 nm. The encapsulated paclitaxel in the PTX-HSA-NPs was calculated using the following equation.

Encapsulation efficiency (EE%) = (concentration of PTX encapsulated/starting concentration of PTX used) × 100

### 4.6. Measuring In Vitro Paclitaxel Drug Release

The *in vitro* drug release was measured by the UV-visible spectrophotometric method as published previously [4,26,27]. In brief, PTX-HSA-NPs were spin concentrated using Amicon centrifugal filters (Cedarlane, Burlington, ON, Canada) with MWCO 10,000 Da. The nanoparticles were re-suspended in 5 mL PBS and placed in a shaker at 37 °C at 120 rpm. At pre-determined time intervals (0, 1, 2, 9, 12, 18, 24 and 48 h), 0.5 mL of the PTX-HSA-NPs solution was withdrawn starting from 0 h for the absorbance measurement at 230 nm and re-substituted with 0.5 mL of fresh PBS. From the absorbance measurements, the cumulative amount of PTX released into the solution at the different time intervals was determined. This study was performed in triplicates.

### 4.7. Cell Viability Due to PTX-HSA-NPs

MCF-7 breast cancer cell lines were received as a kind gift by Dr. Jose Teodoro (Biochemistry, McGill University). The MCF-7 cells were grown in Dulbecco’s Modified Eagle Medium (DMEM). The medium was supplemented with 10% fetal bovine serum (FBS) and 1.5 g/L sodium bicarbonate. The cells were cultured in a humidified incubator at 37 °C with 5% CO_2_. 

To determine the cell viability due to PTX-HSA-NPs, the MTT assay was performed. The MTT assay is a commonly-used colorimetric assay using the MTT for the rapid determination of biomaterial cell toxicity [4]. MCF-7 cells were seeded at an initial concentration of 5,000 cells/mL in 96-well culture plates. After 24 h of incubation in a humidified incubator at 37 °C with 5% CO_2_, the media of the adherent cells was replaced with serum-free culture media and treated with HSA-NPs and PTX-HSA-NPs with a final concentration of 0.2 mg/mL. The cells were further incubated for 24 and 48 h, followed by replacing the treatment medium with 100 μL of fresh cell culture media. To assess the cytotoxicity of nanoparticles, 10 μL of MTT reagent was added to each well containing 100 μL of cell culture medium and incubated in a humidified incubator for 4 h at 37 °C and 5% CO_2_. After incubation, 100 μL of lysis buffer (99.4% dimethylsulfoxide (DMSO) and 0.6% acetic acid) was added to the wells and incubated for another 15 min at room temperature. The absorbance was measured at 570 nm using a Victor3V 1420 Multilabel Counter spectrophotometer (Perkin Elmer, Woodbridge, ON, Canada). For the dose-response study, different concentrations of PTX-HSA-NPs and free PTX were diluted with the cell culture medium and DMSO (less than a 0.5% final concentration of DMSO in solution) to obtain dilutions of 100 μM, 10 μM, 1 μM, 0.1 μM, 0.01 μM and 0.001 μM.

The IC_50_ was calculated using the GraphPad Prism software, version 5.01 (GraphPad Software Inc., La Jolla, CA, USA), following nonlinear regression analysis.

## 5. Conclusions

HSA-NPs are excellent drug delivery systems that can carry a variety of drugs, including paclitaxel. They can be prepared by the emulsion-solvent evaporation method. Various parameters influence the particle size and physical stability of the HSA-NPs. Optimizing these parameters reduced the final particles size to less than 200 nm. The addition of paclitaxel to the HSA solution formed PTX-HSA-NPs. Increasing the PTX concentration in the HSA solution led to an increase in the particle size and higher encapsulation efficiency, without affecting the particle yield. The *in vitro* studies exhibited controlled drug release profiles. The MTT assay on MCF-7 cells incubated with PTX-HSA-NPs showed concentration (PTX)-dependent cytotoxicity. These studies suggest that the optimized PTX-HSA-NPs are capable of an anticancer effect with the optimal therapeutic efficacy and minimum undesirable side effects. Our future studies would include surface modification of PTX-HSA-NPs to make them target specific and further enhance their anticancer effect both *in vitro* and *in vivo*.

## Figures and Tables

**Table 1:** Summary of various parameters optimized for the preparation of HSA-NPs and the resulting nanoparticle size (nm), zeta potential (mV) and polydispersity index (PDI).

**Homogenization Pressure**			
**Pressure (psi)**	**Size (nm)**	**Zeta Potential (mV)**	**PDI**
10,000	337.7 ± 14.8	5.0 ± 12.7	0.26
15,000	248.2 ± 6.6	8.2 ± 16.6	0.27
20,000	254.7 ± 15.5	8.8 ± 8.0	0.24
**Number of Homogenization Cycles**			
**Cycles**	**Size (nm)**	**Zeta Potential (mV)**	**PDI**
6	307.1 ± 9.2	−20.8 ± 6.5	0.25
9	303.7 ± 24.1	−22.2 ± 5.8	0.26
12	242.8 ± 11.7	−18.7 ± 2.9	0.26
15	302.8 ± 17.3	−24.5 ± 3.4	0.32
**Human serum albumin (HSA) Concentration**			
**HSA (mg/mL)**	**Size (nm)**	**Zeta Potential (mV)**	**PDI**
10	216.6 ± 9.8	−14.7 ± 13.7	0.26
20	229.3 ± 9.5	−19.8 ± 2.9	0.29
30	267.9 ± 11.7	−21.4 ± 7.3	0.29
40	343.6 ± 14.4	−18.2 ± 6.5	0.34
**Chloroform Concentration**			
**Chloroform (% *v*/*v*)**	**Size (nm)**	**Zeta Potential (mV)**	**PDI**
3	256.1 ± 22.8	−15.5 ± 6.6	0.30
5	309.4 ± 14.5	−22.4 ± 7.0	0.28
10	280.1 ± 19.1	−26.6 ± 3.9	0.30
20	178.4 ± 6.7	−24.3 ± 6.3	0.24
**Chloroform-Ethanol Concentration (94:6)**			
**CHCl_3_-EtOH (% *v*/*v*)**	**Size (nm)**	**Zeta Potential (mV)**	**PDI**
3	169.1 ± 2.6	−7.9 ± 0.36	0.48
5	152.4 ± 5.1	−9.4 ± 0.3	0.57
10	143.4 ± 0.7	−5.62 ± 0.8	0.43
**Paclitaxel Concentration**			
**PTX (mg/mL)**	**Size (nm)**	**Zeta Potential (mV)**	**PDI**
0.5	170.2 ± 1.4	−17.4 ± 0.5	0.14
1	177.1 ± 2.5	−26.8 ± 3.1	0.09
1.5	207.5 ± 2.4	−12.6 ± 0.7	0.22

**Table 2:** Summary of the yield (%) and encapsulation efficiency (%) values for human serum albumin nanoparticles (HSA-NPs) and paclitaxel human serum albumin nanoparticles (PTX-HSA-NPs) prepared with paclitaxel (PTX) starting concentrations of 0.5, 1 and 1.5 mg/mL.

Type of HSA-NPs with PTX	Yield	Encapsulation Efficiency
PTX-0.5 mg/mL	93.40%	81.80%
PTX-1.0 mg/mL	93.70%	93.94%
PTX-1.5 mg/mL	92.50%	97.96%
PTX-0 mg/mL	93.90%	-
